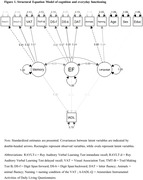# Everyday functioning beyond cognition: Using Structural Equation Modelling to explore relationships between cognitive domains and everyday functioning in Alzheimer’s disease

**DOI:** 10.1002/alz.090660

**Published:** 2025-01-03

**Authors:** Sophie M. van der Landen, Merel C. Postema, Mukrabe E. Tewolde, Frederik Barkhof, Argonde C. van Harten, Charlotte Teunissen, Elsmarieke van de Giessen, Rudolf W. H. Ponds, Wiesje M. van der Flier, Hanneke F.M. Rhodius‐Meester, Sietske A. M. Sikkes

**Affiliations:** ^1^ Alzheimer Center Amsterdam, Neurology, Vrije Universiteit Amsterdam, Amsterdam UMC, Amsterdam Netherlands; ^2^ Amsterdam Neuroscience, Neurodegeneration, Amsterdam Netherlands; ^3^ Alzheimer Center Amsterdam, Neurology, Vrije Universiteit Amsterdam, Amsterdam UMC location VUmc, Amsterdam Netherlands; ^4^ University College London, London United Kingdom; ^5^ Amsterdam Neuroscience, Brain Imaging, Amsterdam Netherlands; ^6^ Amsterdam UMC, Amsterdam Netherlands; ^7^ Neurochemistry Laboratory, Department of Clinical Chemistry, Vrije Universiteit Amsterdam, Amsterdam UMC location VUmc, Amsterdam, North Holland Netherlands; ^8^ Department of Radiology & Nuclear Medicine, Vrije Universiteit Amsterdam, Amsterdam UMC location VUmc, Amsterdam Netherlands; ^9^ Department of Medical Psychology, Amsterdam UMC location Vrije Universiteit Amsterdam, Amsterdam Netherlands; ^10^ Department of Epidemiology and Data Science, Vrije Universiteit Amsterdam, Amsterdam UMC, Amsterdam Netherlands; ^11^ Geriatric Medicine, The Memory Clinic, Oslo University Hospital, Oslo, Oslo Norway; ^12^ Internal Medicine, Geriatric Medicine Section, Amsterdam Cardiovascular Sciences Institute, Vrije Universiteit Amsterdam, Amsterdam UMC, Amsterdam Netherlands; ^13^ Faculty of Behavioural and Movement Sciences, Clinical Developmental Psychology & Clinical Neuropsychology, Vrije Universiteit Amsterdam, Amsterdam Netherlands

## Abstract

**Background:**

Alzheimer’s disease (AD) causes increasing cognitive and functional impairments, and both are therefore important outcome measures for intervention studies. Cognition and everyday functioning are often used interchangeably, yet the extent of their relationship is still unclear. We therefore aim to assess the relationship between different cognitive domains and everyday functioning across the AD spectrum.

**Methods:**

In this cross‐sectional study, we included 613 participants (Mean age ± Standard Deviation = 64 ± 8 years, n = 298(52%) female) from the memory‐clinic based Amsterdam Dementia Cohort, who were amyloid‐β positive based on cerebrospinal fluid. Cognitive functions were assessed using a standardized neuropsychological test battery assessing three domains: memory (Rey Auditory Verbal Learning Test (RAVLT) and Visual Association Test (VAT)), executive functioning (Trail Making Test‐B, Digit Span forward and backward, and letter fluency DAT) and language (animal fluency and naming condition of the VAT). Everyday functioning was assessed by the proxy‐based Amsterdam Instrumental Activities of Daily Living Questionnaire (A‐IADL‐Q). Structural equation modelling (SEM) analysis was performed to examine the relationship between each of the cognitive domains (i.e., composite Z‐scores) as independent variables and everyday functioning as dependent variable, adjusted for age, sex and education. The relations between latent variables (cognitive domains) were calculated while accounting for measurement error in observed variables (cognitive tests).

**Results:**

Of the 613 participants, the majority had a dementia diagnosis (n = 443, 72%), followed by mild cognitive impairment (n = 100, 16%) and subjective cognitive decline (n = 70, 11%). The SEM showed good model fit (CFI = 0.822), and confirmed that the latent cognitive domains (identified as memory, executive functioning and language) were substantially explained by their respective observed indicators with standardized estimates ranging from 0.44 to 0.91. Taking into account interdependencies and unexplained variance, the standardized estimate of memory was significantly related to the A‐IADL‐Q, demonstrating that approximately 28% of variance was explained by memory functioning.

**Conclusion:**

Memory performance was found to be uniquely related to difficulties in everyday functioning, although to a limited extent. Our findings suggest that factors beyond cognition substantially contribute to everyday functioning, highlighting that both have independent value as outcome measures.